# Contrast-enhanced ultrasound of granular cell tumor in breast: A case report with review of the literature

**DOI:** 10.3389/fonc.2022.894261

**Published:** 2022-08-23

**Authors:** Huanyu Wang, Duo Feng, Tianhui Zou, Yao Liu, Xiaoqin Wu, Jiawei Zou, Rong Huang

**Affiliations:** ^1^ Department of Breast and Thyroid Surgery, Huazhong University of Science and Technology Shenzhen Union Hospital, Shenzhen, China; ^2^ Department of Cardiology, Fuwai Hospital Chinese Academy of Medical Sciences, Shenzhen, China

**Keywords:** Contrast-enhanced ultrasound, granular cell tumor, breast tumor, diagnoses, case report

## Abstract

Granular cell tumor is an infrequent, predominantly benign tumor originating from Schwann cells. Granular cell tumor of the breast (GCTB) can simulate breast malignant carcinoma on the clinical assessment. We herein present a rare case of GCTB which recurred in the contralateral breast. We believe the contrast-enhanced ultrasound (CEUS) findings of GCTB have never been described. The high similarity of breast malignant carcinoma makes its differential diagnosis difficult on the clinical and radiological features. In this report, we present the CEUS findings from a rare case of GCTB, explore the possible value of CEUS in differential diagnosis between benign breast lesions and malignant ones, and briefly review the literature.

## Introduction

Granular cell tumor (GCT) was first described by Abrikossoff in 1926, as a benign tumor with a myogenic origin in the breast (1). It is supposed to originate from Schwann cells of the peripheral nerves or their precursors. Hence, the GCT can develop in various organs as the nerves distribute, most commonly in skin or subcutaneous tissue, gastrointestinal tract, head and neck region (2). On pathological examination, the cells mostly contain a unusual granular eosinophilic cytoplasm along with classical nuclei and abounding lysosomes, whose cytoplasm is positive for CD68, S100 protein and neuron-specific endolase (NSE) in the immunohistochemical stain (3). Most of the GCTs are usually benign and solitary, while 2% of them are reported as malignant (4). The granular cell tumor of breast(GCTB) occurs in 5-15% of GCT, which is one of the common sites of GCT (4). GCTB is rarely reported as malignant, as having more than three of the following manifestations: the behave of necrosis, the presence of spindle cells, large nuclear body, high level of mitosis, high rate of nuclei/cytoplasm, and pleomorphism (5).

The GCTB can mimic malignant breast tumors in clinical appearance, making it difficult to differentiate from breast cancer. The conventional ultrasound (US) and mammography (MG) are wildly used to describe breast lumps, but they might describe with low specificity in distinguishing breast granular cell tumor according to previous case reports (6–8). Magnetic resonance imaging(MRI) of the breast is not so effective in diagnosis of GCT as breast malignant tumor (8).

Is there any other preoperative method for diagnosis with more details?

The American College of Radiology (ACR) published the fifth edition of the Breast Imaging-Reporting and Data System (BI-RADS) lexicon in 2003, updated newer version in 2013 (9). According to the ACR BI-RADS US lexicon, lesions with a low chance of malignancy (<2%) are defined as BI-RADS category 3, while category 4 lumps are defined as lumps having a wide range of probability (2-94%). The possibility of malignancy for category 4a lumps is 3-10%, that for category 4b lumps is 11-50%, and that for category 4c lumps is 51-94%. Biopsy of the category 4 lesions should be considered (9). The range of biopsies is extremely wide. Following the conventional ultrasound recommendations for breast, 53.80% (495 of 920 patients) biopsy was unnecessary (10). Studies have revealed that CEUS may improve the diagnostic accuracy of breast lesions, especially for BIRADS 4a and 4b lesions (11, 12).

Hence, we present an uncommon case of GCTB evaluated by CEUS for the first time to date. The objective of this case report is to focus on the imaging features GCTB to reduce misdiagnose of breast malignant tumors.

## Case report

A 28-year-old Chinese woman was admitted at department of Breast and Thyroid Surgery, Huazhong University of Science and Technology Shenzhen Union Hospital, after “feeling a lump on her left breast for about 1 month duration”. Physical examination of the mass on the 6 o’clock position of left breast was found to be about 2×2 cm with blurred borders, poor mobility, and no tenderness on pressure. No obvious enlargement of axillary lymph nodes or no skin thickening was found. She had a lumpectomy for the 10 o’clock lump of her right breast in 2015, The pathological report of her right breast lump was breast granular cell tumor in 2015, whose immunohistochemical staining showed strong S-100 and NSE positivity, with tumor-free margins. Her history of malignant tumors and relevant family was negative.

**Figure 1:**
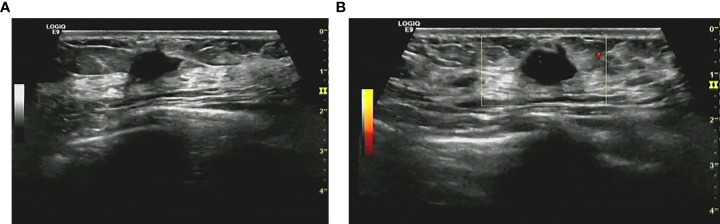
**(A)** Conventional ultrasonography demonstrated an irregular hypoechoic breast lesion without calcification categorized as BI-RADS 4b. **(B)** Only few vascular spots were seen in the lesion.

The lesion was suspicions for malignancy on imaging BI-RADS 4a. There was no distinct finding with MG. Conventional US described a hypoechoic anomalous lump about 13×9 mm, with blurred and angular margins (**Figure 1A**). Color Doppler examination demonstrated 2 vascular spots in the nodule (**Figure 1B**). Strain elastography revealed a mild low strain value in this lesion. The breast MRI was performed to rule out multicentricity or multifocality, as sonographic imaging features suggested malignant tumor. Bilateral breast MRI in a 3.0T system revealed a 12×9mm round lump with a blurred margin in high signal on T2 weighted images on her 5-6 o’clock position of left breast. The mass was intermediate signal on T1 weighted images. Diffusion weighted imaging (DWI), whose b-value was 800 s/mm2, revealed the lump to be of high signal mass. The apparent diffusion coefficient (ADC) of this lesion was about 0.8 × 10–3 mm2/s, while the ADC of contralateral part on the right breast was about 1.7 × 10–3 mm2/s. Dynamic imaging pointed out that the lump to be of high signal mass whose post-gadolinium enhancement imaging revealed an obvious inhomogeneous enhancement with angular margins. The time-signal intensity curve showed type 2. According to its radiology features, a BI-RADS 4c was provided by radiologists.

**Figure 2:**
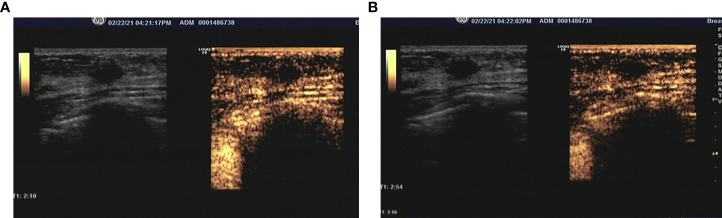
**(A)** During the arterial phase, the lesion was partial enhanced compared with surrounding tissue. **(B)** The micro-bubbles rare filled the lesion still the end of arterial phase, and the margin and shape could still be clear after enhancement.

CEUS was performed for further lesion characterization. The breast was scanned with conventional ultrasound to confirm the mass and determine its most suspicious part and dimension, followed by real-time CEUS imaging. The contrast agent, SonoVue (sulphur hexafluoride microbubbles; Bracco Suisse SA., Plan-Les-Ouates, Switzerland) was given intravenously. Reconstitution powder of SonoVue was mixed with solution as per protocol and 2.4mL was given intravenously, followed by a 10 mL saline flush (0.9% NaCl). Dual-image mode with grayscale and contrast images ensured optimal visualization of the lesion. Compared with the surrounding breast tissue, contrast agent began to wash into the lump partially with iso-enhancement in 10 seconds after injection (**Figure 2A**). In 30 seconds, the micro-bubbles rare filled the lesion, and both the margin and shape could still stay clear after enhancement (**Figure 2B**). CEUS findings of this lesion showed iso-enhancement and synchronous enhancement without enlarged size, while most breast malignant tumors were characterized by fast forward, heterogeneous enhancement, irregular shape, and disordered vessels. Based on the enhancement imagine described by CEUS, two radiologists with over 10-year experience downregulated the lesion as BI-RADS 3.

**Figure 3:**
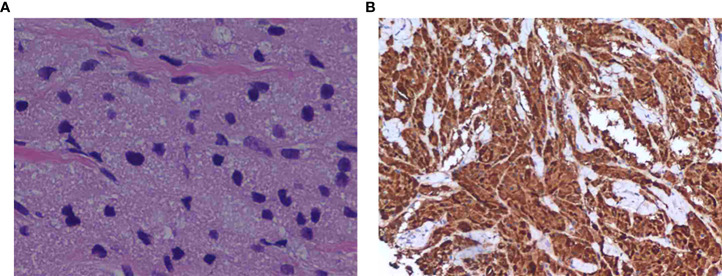
**(A)** Solid nests of tumor cells with coarsely granular cytoplasm. (hematoxylin-eosin, original magnification ×200). **(B)** Staining was positive for the S-100 protein, as shown by brown granules in the cytoplasm of the tumor cells (original magnification, ×200).

Subsequently, the patient underwent a wide local excision to make the margins clean. With histologic examination of the lump, the lump was composed of sheet-forming polygonal cells filled with eosinophilic cytoplasm granules. (**Figure 3A**) Immunohistochemical staining revealed S-100 protein in cytoplasm was positive (**Figure 3B**). The confirmed pathological diagnosis was benign granular cell tumor of breast. In the following 1 year after surgery, the patient recovered well with no recurrence of GCT. This study was approved by the Institute Research Ethics Committee of Huazhong University of Science and Technology Union Shenzhen Hospital and the informed consent was signed by the patient.

## Discussion

A granular cell tumor of breast usually performs as an irregular solid and painless lesion, which may be movable or be fasten to muscle or skin, simulating a breast cancer. Physicians should keep it in mind to distinguish GCTB from breast malignant carcinoma, as misdiagnosis can lead to inappropriate surgery and unexpected psychological and physical trauma to the patient. On the other end, anxiety and fear of malignant neoplasm, which could be a significant psychological trauma for patients, would be brought along with the use of invasive evaluation. How can we make the preoperative diagnosis more precise?

Diagnostic imaging findings of GCTB are variable. On mammography and conventional US, GCTB exhibits atypical morphological characteristics. The GCTB may be described as a round-shaped circumscribed lesion or blurred irregular density on MG. (4) On conventional US, it can be described as a suspicious mass of low echo with irregular or poorly blurred margins, or as a regular circumscribed firm lesion. (13). Elastography would provide an additional information about stiffness as one characteristic. The real-time elastography in conjunction with US has a higher sensitivity. The application of elastography is limited for the small lesions located deeper, as the pressure applied on the skin surface. MG and US remain the standard and important evaluation tools for breast lumps, but the sensitivity of MG or US is <50% for breast cancer. In high-risk Chinese women, the specificity of breast MG alone and US alone were 19.2% and 38.5%, and the sensitivity were 96.1% and 98.6%, respectively. The sensitivity of combined US and MG (50.0%), US followed by MG for triage (46.2%), was significantly higher than MG alone whose sensitivity was 19.2% (p=0.008 and p=0.039). (14). Breast MRI is an important tool in delineating the breast lesions, searching for aggressive flags and existence of other breast lesions. On the bilateral beast MRI, the signal intensity of the lesions on T2 weighted MRI images can range from low to high, compared with the adjacent muscles, which present to enhance homogeneously on T1 weighted MRI images. On the time-course enhancement analysis, the signal intensity may keep increasing throughout the dynamic period. (15) Nevertheless, the cost of breast MRI in both time and ecomonic discourages the patients even some clinicals for annual cancer screening. Besides, in the mainland of China, the cost of MG is 3 times that of ultrasound examination, and ultrasound for breast cancer screening is the lowest cost breast cancer screening system, which is only 18.4% of mammography or 35.6% of combined methods. (14) Nowadays, China’s economic level among regions varies widely. In those comparatively underdeveloped areas, ultrasound alone is preferred for breast cancer screening, while in developed areas, mammography followed by ultrasound for primary screening could be considered.

Neither MG nor conventional US can present details on the microcirculation of breast lesions. CEUS is a novel tool to detect the microvascular. With the application of contrast enhancement, CEUS would provide more information, comparing with the conversional ultrasound. The contrast agent administered intravenously are micro-bubbles filled with gas. They are designed to enhance the details of images and assess vascular structures as intravascular tracers of ultrasonic wave. Several studies have pointed out that the lesions which are enhanced homogeneously or rarely on CEUS tend to be benign. (16, 17) Several studies have revealed the CEUS is helpful in differentiating benign and malignant neoplasms in breast, especially in predicting BI-RADS 4 breast disease. (12, 16, 18) Additionally, some studies showed that CEUS can be effective at downgrading certain BI-RADS 4 masses initially assessed by conventional ultrasound. This pilot studies revealed that the number of benign breast masses recommended for biopsy can be reduced with help of CEUS. (11, 19) Nevertheless, whether breast CEUS is reliable for the diagnosis of rare breast tumor, such as GCTB, should be further studied. More cases of rare breast tumors should be studied, as the pathological types in previous studies were relatively common and limited. Besides, there are some limitations of CEUS needed to acknowledged. It is necessary to standardize the diagnostic criteria of CEUS. The diagnostic accuracy of CEUS might be affected by the patients’ age and the contrast agents. (20) An experienced operator is needed, as the CEUS is a quite operator-dependent technique. CEUS would provide more qualitative (i.e. type of vascularization, perfusion homogeneity and enhancement degree) and quantitative (i.e. region of interest, time intensity curves, time to peak, wash in slope, peak intensity, area under cruve) information. (19) The analysis of these information needs to be studied further to improve the diagnostic accuracy.

## Conclusion

GCTB is similar to breast malignant neoplasm and may exhibit suspicious morphological analysis on all radiological evaluations as the irregular behavior. Features on CEUS may suggest a benign tumor to decrease the biopsy rate. Otherwise, it is impossible to confirm an accurate diagnosis of GCTB clinically and radiologically. The diagnosis of the GCT is dependent on its histopathological examination. Local excision with wild margins requiring careful evaluation is recommended. Radical local excision with negative margins is recommended in all cases of GCT, as positive margins are contributed to recurrence of the neoplasm. (21) There was no adjuvant therapy recommended after excision. However, follow-up for 10 years after surgery is strongly recommended, even with negative resection margins. CEUS is an efficient, feasible, non-irradiating, accessible imaging technique that assesses qualitative characteristics and quantitative parameters of breast lesions. The application of CEUS would be helpful in decreasing the percentage of breast biopsies. With the use of CEUS, the breast cancer screening system would be more effective. Moreover, the reporting and data system of CEUS for beast lesions should be developed and standardized.

## Data availability statement

The original contributions presented in the study are included in the article/supplementary material. Further inquiries can be directed to the corresponding author.

## Ethics statement

Written informed consent was obtained from the individual(s) for the publication of any potentially identifiable images or data included in this article.

## Author contributions

Conceptualization: HR and FD. Treatment decision-making and discussions: WH, FD, ZT, LY, WX; ZJ and HR. Data collection and analysis: LY, FD, ZT, WX and ZJ. Manuscript writing: WH and ZJ. Final approval of manuscript: ZT, FD and HR. All authors contributed to the article and approved the submitted version.

## Acknowledgments

We sincerely thank the patient for her willingness to share all her experiences and feelings with the public.

## Conflict of interest

The authors declare that the research was conducted in the absence of any commercial or financial relationships that could be construed as a potential conflict of interest.

## Publisher’s note

All claims expressed in this article are solely those of the authors and do not necessarily represent those of their affiliated organizations, or those of the publisher, the editors and the reviewers. Any product that may be evaluated in this article, or claim that may be made by its manufacturer, is not guaranteed or endorsed by the publisher.
